# Evaluating the Effect of Ramadan Fasting on Muslim Patients with Diabetes in relation to Use of Medication and Lifestyle Patterns: A Prospective Study

**DOI:** 10.1155/2014/308546

**Published:** 2014-11-11

**Authors:** Melanie Yee Lee Siaw, Daniel Ek Kwang Chew, Rinkoo Dalan, Shaikh Abdul Kader Kamaldeen Abdul Shakoor, Noorani Othman, Chor Hui Choo, Nur Hidayah Shamsuri, Siti Nurhana Abdul Karim, Sui Yung Chan, Joyce Yu-Chia Lee

**Affiliations:** ^1^Department of Pharmacy, Faculty of Science, National University of Singapore, Block S4, 18 Science Drive 4, Singapore 117543; ^2^Department of Endocrine and Diabetes, Tan Tock Seng Hospital, 11 Jalan Tan Tock Seng, Singapore 308433; ^3^Department of Pharmacy, Tan Tock Seng Hospital, 11 Jalan Tan Tock Seng, Singapore 308433

## Abstract

*Objectives*. This study aimed to examine the effect of Ramadan fasting on HbA1c in Muslim patients with type 2 diabetes. The incidence of hypoglycemia and glycemic changes in relation to the adjustment of doses of antidiabetic agents, diet, and physical activity during Ramadan was also evaluated. *Methods*. This was a prospective study conducted in an outpatient endocrine clinic. A set of questionnaires was administered to Muslim patients with diabetes who fasted for ≥10 days. Those who were hospitalized for diabetic ketoacidosis or severe hypoglycemia a month prior to Ramadan or were given short-term corticosteroid therapy were excluded. The patients' responses and clinical outcomes from the clinic database were collected before, during, and after Ramadan. *Results*. A total of 153 participants completed the study. The mean HbA1c improved from 8.9% before Ramadan to 8.6% during Ramadan (*P* < 0.05). Although diet and physical activity did not contribute to changes in glycemia, a significant improvement in HbA1c was observed in patients who had adjustments made to their doses of antidiabetic agents during Ramadan (*P* < 0.001). In addition, their rate of hypoglycemia was minimal. *Conclusions*. Ramadan fasting appeared to improve glycemic control, especially in those whose doses of antidiabetic agents were adjusted during Ramadan.

## 1. Introduction

Ramadan is a holy month in the Islamic calendar during which Muslims all over the world observe a fast between dawn and sunset. During Ramadan, Muslims who are ill or have health conditions that may deteriorate upon fasting, including patients with diabetes, are exempt [1]. However, fasting during Ramadan is highly regarded as a form of religious practice, and thus many devoted Muslim patients insist on fasting despite being advised not to by their healthcare providers. It has been estimated that 40–50 million individuals with diabetes fast during the month of Ramadan [2].

Ramadan fasting entails major changes in dietary patterns and frequency. These changes could potentially induce metabolic alterations in both healthy and diseased Muslims [3, 4]. Despite taking fewer meals, this practice is usually compensated by ingesting large amounts of sugary food and drinks that are high in carbohydrates and fats, especially when breaking the fast [5]. The overall calorie consumption of individuals with type 2 diabetes has been reported to increase during Ramadan [6, 7]. Moreover, the doses of antidiabetic agents are often adjusted at this time to reflect the change in lifestyle during Ramadan. One study showed that, among diabetic patients whose doses of oral hypoglycemic agents (OHA) were modified during Ramadan, 58% had changed the timings of the administration of their medications [8].

During Ramadan, it has also been reported that physical activities, especially exercising, tend to decrease from a fear of feeling too weak [9]. Patients with diabetes have been advised by healthcare providers to avoid excessive physical activity during Ramadan, because the practice of fasting may increase the risk of hypoglycemia, especially a few hours before the sunset meal. However, one study reported that appropriate levels of physical activity during fasting did not interfere with tolerance to physical exercise [10].

Furthermore, it has also been postulated that the act of fasting may increase the risk of poor glycemic control, which raises questions about the safety of Ramadan fasting in patients with diabetes. During Ramadan fasting, the decrease in blood glucose levels triggers compensatory mechanisms in the body of healthy individuals which cause a reduction in insulin secretion or the breakdown of stored glycogen to prevent hypoglycemia [11]. However, in patients with diabetes, this regulation is compromised due to either a dysfunction of insulin secretion or sensitivity or occasionally both [12]. One epidemiological study reported that the risk of severe hypoglycemia increased sevenfold during the month of Ramadan in patients with diabetes [2]. However, another study reported that Ramadan fasting was safe and did not significantly increase the incidence of hypoglycemic events [13].

The primary objective of this prospective study was to evaluate the effect of fasting during Ramadan on the glycemic control of Muslim patients with type 2 diabetes by examining the changes in levels of HbA1c before, during, and after Ramadan. In addition, the incidence of hypoglycemia and changes in glycemic control in relation to the adjustment of doses of antidiabetic agents, diet, and physical activity during Ramadan were examined.

## 2. Methods

### 2.1. Study Design and Site

This was a prospective study conducted in an outpatient endocrine clinic located within a tertiary hospital in Singapore between June and November 2012; Ramadan took place from July 21 to August 18, 2012. In Singapore, Muslims account for 14.7% of the population [14], and, during Ramadan, Muslims fast for 30 days [15]. The study, which was approved by the Institutional Review Board, comprised of data collection from the institutional database and the administration of a set of questionnaires by trained interviewers. Eligible patients were screened and approached to determine their interest in participating in this study a month before Ramadan. Upon signing the consent form, a set of questionnaires was administered to study patients by interviewers before, during, and after Ramadan. Data on pertinent clinical outcomes were also collected at the three time periods.

### 2.2. Inclusion and Exclusion Criteria

All Muslim patients over 21 years of age with type 2 diabetes who fasted for at least 10 days [16] during the month of Ramadan were included in this study. The patients who reported having been hospitalized for diabetic ketoacidosis or severe hypoglycemia a month prior to Ramadan or those who received short-term corticosteroid therapy were excluded from the study. In addition, the patients who were unable to comprehend the questionnaire were excluded from the study.

### 2.3. Questionnaire

The questionnaire used in this study was adapted from a 2011 prospective study after consent was obtained from the authors [8]. The questionnaire comprised a sociodemographic section, which included age, gender, level of education, employment status, body mass index, health status, and duration of diabetes, and a section on the types of antidiabetic agents used and dosing regimens, the patients' perception of their daily physical activities, and their dietary patterns. For the questions relating to diet and physical activity, changes in these parameters during Ramadan were self-reported by the patients based on a response of more, less, or unchanged.

Minor amendments were made to questions related to sociodemographics and diet to suit the local context. The questionnaire was translated into the Malay language commonly spoken by the Muslim population in Singapore. Questionnaires were administered to patients in their preferred language by the trained, bilingual interviewers and took approximately 30 minutes to complete.

Outcome measures, such as HbA1c, and responses to the questionnaire were collected before, during, and after Ramadan. HbA1c is a surrogate marker, commonly used in numerous studies related to Ramadan fasting, which reflects glycemic control in Muslim patients; it gives a more reliable reflection of the average plasma glucose concentration over 8–12 weeks [17]. In this study, the incidents of hypoglycemia were classified as minor or major. Minor hypoglycemia was defined as an event that can be self-managed by patients irrespective of the severity of the symptoms, while severe hypoglycemia was defined as an event that requires the assistance of a third party to effect treatment [18]. The study patients were also asked if they had experienced any symptoms of hypoglycemia during Ramadan fasting [8].

### 2.4. Statistical Analysis

The data are presented as means ± standard deviations (SD) for continuous variables and as percentages for categorical variables. The outcome measure, HbA1c, was analyzed between two time periods using a linear mixed model; this was followed by pairwise comparisons with Bonferroni adjustment. Diet and physical activities were compared with HbA1c as binary outcomes in logistic regression. All of the analyses were adjusted for age, gender, BMI, level of education, total comorbidities, duration of diabetes, and employment and health status. After multiplication by the number of repeated analyses where appropriate, a two-tailed *P* < 0.05 was considered to be significant. The computations were performed using SPSS for Windows, version 19.0 (SPSS Inc., Chicago, IL, USA).

## 3. Results

Of the 251 patients recruited, 98 (39%) were excluded from the analysis due to incomplete responses. At the completion of the study, 153 patients with an average of 26 fasting days had been followed up throughout the entire study to give a response rate of 61%. Their mean age was 56.7 ± 9.1 years and 62.7% of the participants were women (*n* = 96) (Table 1). A large majority (*n* = 81; 52.9%) of the study subjects had at least a 12th grade education. However, more than half of the participants were unemployed (*n* = 90; 58.8%). With regard to diabetes management, 150 (98%) were taking antidiabetic agents of which 72 (47.1%) were on a combination regimen of OHA and insulin. Metformin was the most commonly prescribed OHA (*n* = 119; 92.2%), followed by sulfonylurea (*n* = 70; 54.3%) and dipeptidyl peptidase 4 inhibitors (*n* = 11; 8.5%). The majority were given basal insulin concurrently with mealtime insulin (*n* = 61; 65.6%) while the remainder were either given basal insulin (*n* = 31; 33.3%) or mealtime insulin alone (*n* = 1; 1.1%) (Table 2).

### 3.1. Effect on HbA1c

**Figure 1:**
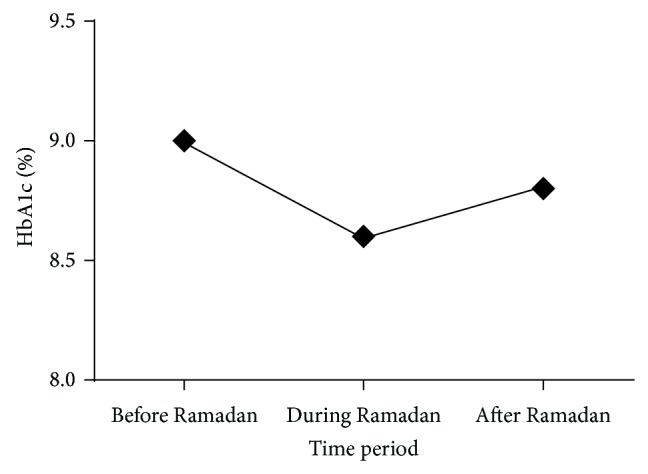
Trends of change in mean HbA1c.

Mean HbA1c improved from 8.9 ± 2.0% before Ramadan to 8.6 ± 1.8% during Ramadan (*P* < 0.05). However, this improvement was not sustained after Ramadan. An increase of 0.2% in mean HbA1c was observed between Ramadan and after Ramadan (*P* > 0.05) (Figure 1).

### 3.2. Dose Adjustment in relation to Glucose Control

During Ramadan, the most common method of dose adjustment made by the OHA users was a reduction in total daily dose (*n* = 55; 73.3%). The other methods of dose adjustment were cessation of OHA (*n* = 9; 12.0%), increased night-time dose alone (*n* = 5; 6.7%), increased total daily dose (*n* = 3; 4.0%), and additional OHA (*n* = 3; 4.0%). In this study, the three most commonly adjusted OHAs were metformin, glipizide and its equivalents [19, 20], and sitagliptin with average dose reductions of 810.4 ± 632.2 mg, 4.5 ± 6.9 mg, and 16.7 ± 57.7 mg, respectively, during Ramadan. Among the insulin users, the total daily insulin dose was reduced by 39 (60.9%) patients and stopped by five (7.8%) patients. Of the remaining patients who adjusted their insulin therapy, two (3.1%) took additional insulin, six (9.4%) increased their total daily dose, and 12 (18.8%) increased their night-time insulin dose alone. Overall, the average dose adjustments of insulin during Ramadan for mealtime and basal insulin were −11.8 ± 13.6 units and −8.1 ± 10.4 units, respectively.

**Figure 2:**
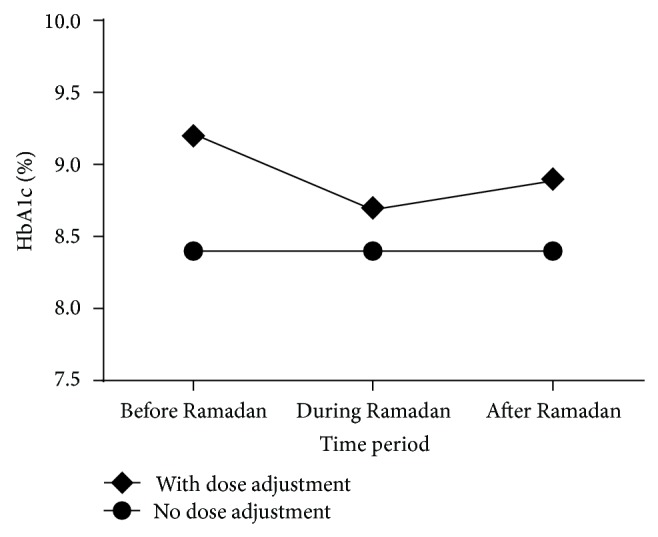
Trends of change in mean HbA1c in relation to adjustment of the dose of antidiabetic agents.

An improvement in mean HbA1c from 9.2 ± 1.9% before Ramadan to 8.7 ± 1.6% during Ramadan was observed in patients who made dose adjustments during Ramadan (*n* = 104; *P* < 0.001). In patients who did not make any dose adjustments during Ramadan, no significant improvement in HbA1c was observed (*n* = 49; *P* > 0.05) (Figure 2).

### 3.3. Diet and Physical Activity in relation to Glucose Control

During Ramadan, 99 (64.7%) participants reported a reduction in dietary intake, 45 (29.4%) reported an unchanged dietary intake, and nine (5.9%) reported an increased dietary intake. With regard to physical activity, the majority (*n* = 96; 62.7%) reported an unchanged level of activity while 49 (32%) reported less activity and eight (5.2%) increased their level of activity during Ramadan. These perceived patterns of diet and physical activity were, however, found to have no significant association with the improvements in mean HbA1c during Ramadan fasting (*P* > 0.05).

### 3.4. Incidents of Hypoglycemia

In this study, no major hypoglycemic events were reported by the patients. However, 39 (25.5%) patients reported having experienced minor hypoglycemia during Ramadan and of which 17 (43.6%) were on combination regimen of OHA and insulin, 14 (35.9%) were on OHA alone, and 8 (20.5%) were on insulin alone. Commonly reported hypoglycemic symptoms included dizziness, tremors, increased sweating, extreme hunger, increased heart rate or palpitation, and increased frequency of headaches, nausea, confusion, and mood changes.

## 4. Discussions

This was the first prospective study to observe the changes in glycemia before, during, and after Ramadan fasting and also provided a comprehensive understanding on the effects of lifestyle modification and dose adjustment on glycemia during Ramadan fasting.

In this study, a significant reduction of 0.3% in mean HbA1c was observed during Ramadan fasting. Although an improvement in glycemia was found, a 25.5% rate of minor hypoglycemia occurred in these patients. While any form of hypoglycemic event is undesirable in the management of diabetes, this rate is nevertheless lower than or comparable with other studies conducted in nonfasting patients with type 2 diabetes. A cross-sectional study by Miller et al. [21] reported hypoglycemia rates of 12% for patients on diet alone, 16% for patients on OHA, and 30% for patients on insulin.

Some studies have shown that people who received adjustments to antidiabetic drug doses during Ramadan achieved better glucose control [22, 23]. A study reported by Mafauzy [22] showed that those with adjustments to their dose of OHA experienced a larger reduction in their level of fructosamine (−21.9 ± 4.8 *μ*mol/L) than those with no adjustment to their OHA dose (–6.7 ± 5.6 *μ*mol/L) during Ramadan (*P* = 0.01). In another study by Hui et al. [23], patients who were switched to faster-acting insulin had an improvement of 0.48% in HbA1c (*P* = 0.0001) while those who were maintained on the same insulin regimen had an improvement of only 0.28% in HbA1c (*P* = 0.007) during Ramadan. Similarly, this study also showed a larger reduction in HbA1c among those with adjustments to their dose of antidiabetic agents. Indeed, the attempt to mimic the physiological secretion of insulin by adjusting the dose during Ramadan may have contributed to the improvement in glycemia.

In our study, diet and physical activity were not found to be related to a reduction in HbA1c during Ramadan. This lack of an association could be explained by examining the pattern of dietary intake and the level of physical activity in our study population. During Ramadan, the majority of patients reported that their dietary intakes were reduced but this surprisingly did not contribute to a reduction in glycemia. One possible reason could be that these patients had perceived a reduction in their dietary intakes due to the reduction in meal frequency but that their daily caloric intakes may have remained the same throughout Ramadan. A study conducted by Haouari-Oukerro et al. [24] showed that overall caloric consumption remained unchanged even though the number of meals was reduced to twice daily during the fasting month. In addition, most of our patients did not report any change in their physical activities during Ramadan; hence, the effect of physical activity on glycemia may be very small in this population. Furthermore, our findings were also observed in several studies in which glycemic control was not associated with physical activity [25, 26].

This study had several limitations. First, it relied on patient self-reporting for certain information such as dietary pattern, physical activity, the incidence of hypoglycemia, and the management of diabetes. Recall bias may have affected the quality of our data but, whenever possible, we also compared the patients' responses with objective data found in patient case notes and the clinical database. Second, we had to exclude 39% of the patients recruited due to our stringent criteria of accepting only questionnaires with a 100% response rate. However, based on the drop-out rate of 32–44.2% of similar studies [27–29], our response rate appears to be reasonable.

Future studies should compare various types of OHA in relation to their risks for hypoglycemic events during Ramadan fasting. This will allow patients and clinicians to closely monitor antidiabetic agents that may be more likely to contribute to hypoglycemic symptoms during Ramadan. In addition, the quality of pre-Ramadan structured education on diet, physical activity, and dose adjustment of medication should also be evaluated to ensure effective and safe diabetes management and positive outcomes.

## 5. Conclusions

Ramadan fasting was observed to improve glycemic control among our patient population with minimal hypoglycemic events. The improvement in HbA1c was more notable in patients whose medications were adjusted in an attempt to mimic the physiological secretion of insulin throughout the fasting period. During Ramadan, clinicians should individualize the dosing of antidiabetic agents especially in patients who intend to fast.

## Figures and Tables

**Table 1:** Patient demographics and attributes.

Patient characteristics	All (*n* = 153)
Gender	
Male	57 (37.3)
Female	96 (62.7)
Age (years)	56.7 ± 9.1
BMI (kg/m^2^)	30.4 ± 6.7
Education level	
No formal education	11 (7.2)
1st to 6th grade	52 (34.0)
7th to 12th grade	81 (52.9)
College/university	9 (5.9)
Employment status	
Employed	63 (41.2)
Unemployed	90 (58.8)
Average number of fasting days	26 ± 5
Duration of diabetes (years)	13.2 ± 9.1
Comorbidities^a^	4 ± 2
Antidiabetic medication	
No medication	3 (2)
OHA^b^ alone	57 (37.3)
Insulin alone	21 (13.7)
OHA^b^ and insulin	72 (47.1)

Data are presented as number (%) or means ± SD.

^a^The three most common comorbidities were hypertension, hyperlipidemia, and neuropathy.

^b^Oral hypoglycemic agent.

**Table 2:** Mean daily dose of oral hypoglycemic agents and insulin at baseline.

Drug class^a^ (number of patients)	Metformin (*n* = 119)	Glipizide^b^ (*n* = 70)	Sitagliptin^c^ (*n* = 10)	Mealtime insulin (*n* = 62)	Basal insulin (*n* = 92)

Mean daily dose (mg/unit)	1795.0 ± 698.4 mg	15.9 ± 9.4 mg	65.0 ± 24.2 mg	30.7 ± 24.1 units	38.1 ± 27.5 units

Values are represented as means ± SD.

^a^Other oral hypoglycemic agents not included in the table were acarbose (*n* = 3; 100 ± 0 mg daily), pioglitazone (*n* = 2; 22.5 ± 10.6 mg daily), and repaglinide (*n* = 1; 1 ± 0 mg daily).

^b^Glipizide-equivalent dose for sulfonylurea was calculated for tolbutamide, gliclazide, and glyburide [19, 20].

^c^One participant was given s-linagliptin (5 mg daily).
